# The impact of changing nonimmigrant visa policies on international students’ psychological adjustment and well-being in the United States during the COVID-19 pandemic: a qualitative study

**DOI:** 10.1186/s12889-022-14698-1

**Published:** 2022-11-30

**Authors:** Chulwoo Park, Shannon Shimada

**Affiliations:** Department of Public Health and Recreation, San José State University, 1 Washington Sq, San Jose, CA 95192 USA

**Keywords:** International student, July 6, 2020 Policy Directive, 2020 presidential election, Social adjustment, Mental health, Psychological well-being, Nonimmigrant visa, Permanent residency, In-depth interview, Qualitative study

## Abstract

**Background:**

Since the Immigration and Nationality Act of 1952, the number of international students in the United States had been gradually increasing. However, the total numbers have begun to decrease since 2019–2020 school year due to the Trump administration's policy and COVID-19. Still, little is known about how international students’ psychological adjustment and well-being have been affected by changing nonimmigrant visa policy and the COVID-19 pandemic.

**Methods:**

We conducted a total of 34 online semi-structured in-depth interviews with international students from 18 countries of origin studying in the San Francisco Bay Area, California. More than 60% of the participants (21 out of 34) were aged 21 to 25. Among our 34 participants, gender and 18 were male and 16 were female, and 19 were undergraduate students and 15 were master’s students. The majority of the participants were first-generation college students (22/34, 64.71%). Verbatim transcription was done for all interviews. NVivo was used for both deductive and inductive approaches to the qualitative analysis.

**Results:**

Overall, the recent political climate negatively impacted participants’ psychology of adjustment and well-being. July 6, 2020 Policy Directive for international students caused severe uncertainty about whether they can continue studying in the United States. There were many resources or services needed to overcome this period, such as extended mental and emotional support from the counseling services as well as financial and informational support from the international student office and university. Although international students had the benefit of the university's food assistance program, they were not eligible to receive any external support outside of the university and financial aid at the local and federal levels. Whether maintaining F-1 visa status was one of their major concerns. Due to COVID-19, job opportunities were limited, which made international students difficult to obtain Curricular Practical Training (CPT) and secure a job in the United States within the 90-day unemployment limit of Optical Practical Training (OPT). H-1B visa and permanent residency were other challenges to go through, but participants saw positive perspectives from the Biden administration.

**Conclusions:**

Uncertain policy changes due to COVID-19 and presidential transitions impacted international students’ psychological well-being and adjustment. International students are important populations in the United States who have supported jobs that are high in demand and economically contributed to the United States. It is expected that future policies at various levels support international students’ life and improve their health equity and mental health.

## Background

Since the Immigration and Nationality Act was enacted in 1952, the F-1 visa program has allowed foreign nationals to study full-time at educational institutions in the United States [1]. Prestigious educational institutions have attracted high-skilled, talented foreign students, which made the United States the prominent global hub for academic training [2]. The enrollment numbers of international students had gradually increased since 1950s, however, the 2019–2020 school year showed the first decline in the total number of international students in the United States [3, 4]. During the 2019–2020 school year, a total of 1,075,496 international students were enrolled at U.S. colleges and universities, which represented 5.5% of the total U.S. student population [3]. However, due to the COVID-19 pandemic and unwelcoming policy for international students, the total enrollment declined to 914,095 during the 2020–2021 school year, a 15% decrease in annual change compared to the previous year [3]. New international student enrollment plummeted to 145,528 during the 2020–2021 school year, a 45.6% decrease from the last year [5].

**Fig. 1:**
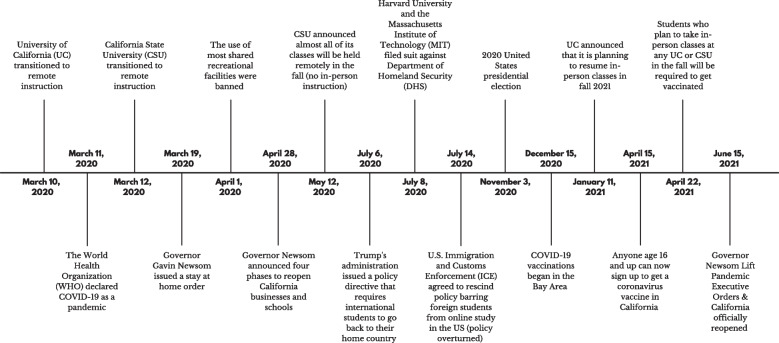
The timeline of policy changes that affected international students during March 10, 2020—June 15, 2021

The 2020–2021 academic year was the most challenging period for international students due to the COVID-19 pandemic and change in the political climate. While the psychological distress of COVID-19 has had a negative impact on the mental health of U.S. college students [6–8], international students have additionally dealt with feelings of isolation and limited access to mental health services [9–11]. In addition to the stress caused by COVID-19, international students had to suffer the July 6, 2020 Policy Directive issued by the Trump administration [12]. When COVID-19 pushed post-secondary institutions to switch the mode of delivery to virtual learning, the Trump Administration issued the July 6, 2020 Policy Directive that international students are not permitted to remain in the United States if taking fully online classes in the fall 2020 semester [12]. However, the administration rescinded this new guidance in response to various lawsuits filed by more than 20 states, the District of Columbia, and two dozen universities [13, 14]. When COVID-19 was declared a global pandemic in March of 2020 [15], almost all post-secondary U.S. educational institutions closed in-person classes and switched them to remote learning [16]. International students had to adjust to the new learning environment, which is virtual learning, and experience a lack of support with the risk of deportation [16]. The result of the 2020 U.S. presidential election gave new hope to international students; the Biden administration released the statement about “A Renewed U.S. Commitment to International Education” [17] and NAFSA provided the recommendations about “Rebuilding and Restoring International Education Leadership” for the Biden administration [18]. Figure 1 demonstrates the timeline of policy changes due to COVID-19 that have affected international students, the period between transitioning to remote classes and returning to normalcy in California as an example [12, 19–34].

International students have positively contributed to the U.S. economy and schools. According to NAFSA, international students at U.S. colleges and universities grew the economy by contributing $28.4 billion and supporting 306,308 jobs during the 2020–2021 academic year [35]. International students boost revenues for U.S. colleges and universities, so the growth of educational institutions and the international students’ enrollment is interdependent [36, 37]. In addition, international students can bring diverse cultural perspectives, which would enrich campus environment. Students’ experiences with diversity are positively associated with their learning, preparation for participating in diverse society and workforce, ability of working with others, and respect for diversity [38, 39]. Moreover, increasing intercultural communication competence on campus is expected to broaden the scope of teaching and research perspectives that eventually benefits all students, faculty, and the public. While many studies reported college students’ mental health during COVID-19 [6–8, 40–42], little is known about how the recent political change and COVID-19 have impacted international students. What remains unclear is how their education has been interrupted, how their mental health status has been changed, and how they need to be supported to continue their education in the United States. The overarching purpose of this study was to address the gaps in knowledge of international students who have been underserved by the changes in nonimmigrant visa policy and COVID-19. Reflecting the NAFSA’s data analysis that California has the largest number of international students with the biggest economic contribution from them, followed by New York and Texas [43], this study sought to explore the impact of the COVID-19 outbreak and the recent political atmosphere–the July 6, 2020 Policy Directive, governmental administration transition, and changes in nonimmigrant visa policy–on international college students studying in California, United States.

## Methods

### Study design and participants

A qualitative study was conducted from April to May 2021. We used a convenience and purposive sampling method to recruit participants who are conveniently accessible to the authors and who shared particular characteristics defined for a purpose relevant to this study. To announce our study for recruitment of international students, we reached out to the international student office, student organizations, academic and honorary organizations, cultural and religious organizations, financial aid and scholarship office, and dormitories at one of the universities in the San Francisco Bay Area. Qualtrics^xm^ (Qualtrics International Inc., Provo, UT) was used to collect the participants' demographic information. At the beginning of the online survey, we asked about the willingness to participate in an in-depth interview. We used Zoom (Zoom Video Communications, Inc., San Jose, USA) to conduct online in-depth interviews in compliance with the social distancing policy in the San Francisco Bay Area.

The inclusion criteria for participants in this study were international students aged 18 years and older studying in the San Francisco Bay Area, California. They also had to maintain the status of full-time student status and registered for classes for at least one semester, either Spring 2020, Fall 2020, or Spring 2021. The demographic information of the participants is shown in Table 1. The majority of participants were from Asia (32/34, 94.12%). More than half of the participants (21/34, 61.76%) were 21–25 years of age. There was a good balance of gender (male: 18/34, 52.94%, female: 16/34, 47.06%) and of program type (undergraduate: 19/34, 55.88%, master: 15/34, 44.12%). Almost half of the participants' family income in the past 1 year (15/34, 42.42%) was less than $25,000, and about one-third of the participants (10/34, 30.3%) did not know or were not sure about their family income. About two-thirds of the participants (22/34, 64.71%) were first-generation college students.

**Table 1:** Demographic statistics of participants aged 18 or older (*N* = 34)

**Gender, *n* (%)**			**Years as an international student in the US, *n* (%)**		
Male	18	52.94%	Less than 1 year	9	29.41%
Female	16	47.06%	1–2 years	11	32.35%
**Age, *n* (%)**			2–5 years	12	35.29%
18–20	6	17.65%	5 or more years	2	5.88%
21–25	21	61.76%	**Place of living, n (%)**		
26–29	5	14.71%	Home- with family	10	29.41%
30 and older	2	5.88%	Home- with friends	2	5.88%
**Country of origin, *n* (%)**			Off campus shared living	12	35.29%
Africa	1	2.94%	Off campus alone	4	11.76%
**Asia**			On campus shared living	5	14.71%
East Asia	6	17.65%	On campus alone	1	2.94%
South Asia	9	26.47%	**Family income in the past 1 year, *n* (%)**		
Southeast Asia	13	38.24%	< $25,000	15	42.42%
Western Asia	4	11.76%	$25,000—$49,999	5	15.15%
Europe	1	2.94%	$50,000—$74,999	1	3.03%
**Program type, n (%)**			$75,000—$99,999	2	6.06%
Undergraduate	19	55.88%	$100,000—$149,999	0	0.00%
Master	15	44.12%	≥ $150,000	1	3.03%
**First-generation college student, *n* (%)**			Don’t Know/Not sure	10	30.30%
Yes	22	64.71%	**Have any family members/relatives in the US, *n* (%)**		
No	12	35.29%	Yes	12	35.29%
**First enrollment as an international student in the US, n (%)**			No	22	64.71%
Before Spring 2020	21	61.76%	**Family members/relatives studied in the US before you, *n* (%)**		
Spring 2020	5	14.71%	Yes	14	41.18%
Fall 2020	3	8.82%	No	20	58.82%
Spring 2021	5	14.71%			

### Qualitative data collection and analysis

A total of 34 participants attended a semi-structured in-depth interview through Zoom. We considered that data saturation was reached at 34th participant’s interview because there was no additional new data found, which was not necessary for further data collection [44]. The average duration of the interview was 50 min (minimum: 20 min, maximum 101 min). Each participant received an incentive, a $20 gift card as a token of appreciation, after the interview. To prepare the interview questionnaire, the authors researched for the exact timeline of the policy changes that may directly or indirectly affect international students, from school’s transition to remote instruction (Mar 10, 2020) to official reopening of California (June 15, 2021) (Fig. 1). Then, we set the COVID-19 pandemic, July 6, 2020 Policy Directive, and change of administration as main incidents or events during that timeline that need to be focused for the interview. In addition, we included the topics related to participants’ active attitudes, such as their coping strategies in reaction to those incidents/events and their plan for the future after graduation from the higher educational institution in the United States. The interview questionnaire consisted of 24 open-ended questions from 6 topic areas for the entire interview: 1) experience before COVID-19 (4 questions), 2) experience during COVID-19 (4 questions), 3) July 6, 2020 Policy Directive (5 questions), 4) coping strategies (5 questions), 5) thoughts on the change of administration (2 questions), and 6) future (4 questions). Among those questions, we focused on 17 questions from 5 topic areas related to the purpose of this study: the impact of the recent political climate that has affected international students’ life (Table 2).

**Table 2:** The interview guide

Topic areas	In-depth interview key questions
Experience during COVID-19	1. How has your living situation changed since the COVID-19 began? (location, positively, negatively, etc.)
	2. How has your academic performance been impacted by COVID-19? If it has been negatively or positively impacted, what are the reasons for the change?
	3. What are your thoughts on continuing your education and your ability to finish your degree in the United States especially during the COVID-19 pandemic?
	4. Can you share any other challenges being an international student due to COVID-19?
July 6, 2020 Policy Directive	1. How did you find out about the July 6, 2020 Policy Directive? How did it make you feel and what are some thoughts that went through your head when you heard about it?
	2. What actions did you take after hearing about the July 6, 2020 Policy Directive?
	3. What kind of resources/information was available to you at that time?
	4. After the policy directive was overturned, what were your thoughts, feelings, etc. Did your plans in the United States change?
	5. What other resources or services would you like to have that have not been provided by your university?
Thoughts on change of administration	1. How did the 2020 presidential elections affect your academic performance?
	2. How do you feel about the change in governmental administration? What expectations do you have from the new administration for international students?
Coping strategies	1. What type of support system do you have? (Academic, family, friends, etc.)
	2. What type of support do you think is helpful for international students to be successful in the US?
Future	1. What is your dream? What is your career goal in the future?
	2. What motivates you as a international student to achieve your future goals?
	3. Overall, what role can university campuses or institutions play in making academic/education experience in the US equitable for international students?
	4. Any additional comments or thoughts? Anything that you like to share or would like to add to earlier questions about your experience as an international student in the U.S.?

Each of the audio-recorded interviews was transcribed verbatim in a separate word document. Then, we used QSR NVivo software (QSR International, Pty, Ltd, Doncaster, Australia) to analyze 34 verbatim transcripts excluding personal identifiers. Two approaches for coding were used; deductive coding was first used from a predetermined questionnaire, then further transcript analysis was followed by inductive coding from newly uncovered information.

### Ethical considerations

This study received exempt registration from San José State University’s Institutional Review Board (IRB). In addition, a waiver of signed consent was approved by San José State University's IRB. Consent notices for an in-depth interview and an online survey were provided at the beginning of the Qualtrics survey to participants. To ensure the confidentiality of the participants, we informed them before the beginning of the interview that they can choose to use their nickname or pseudonym and switch off the video in Zoom. To preclude identification, only participant number (e.g., #1—#34) was included at the end of each quote in the results section.

## Results

**Fig. 2:**
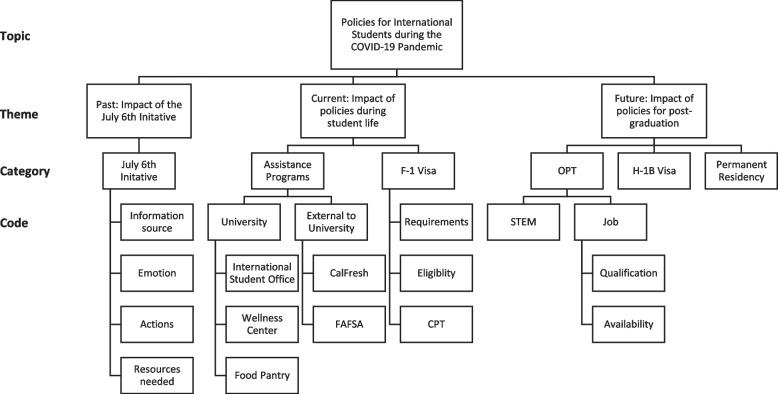
Coding tree for international students’ policies

We categorized themes with nonimmigrant visa policy [45] in chronological order as compared to the timing of the interview period. First, we asked about the impact of the July 6, 2020 Policy Directive that might greatly impact international students' plans to study in the United States. Second, we wondered how current resources and policies, such as university assistance programs, F-1 visa, and OPT/CPT, have impacted international students during the COVID-19 pandemic. Lastly, our participants shared their dream and career goal in the future aligning with the relevant visa policies, such as H1B visa, and permanent residency. Our thematic analysis extracted the following time-based three policy themes: 1) past: impact of the July 6, 2020 Policy Directive, 2) present: impact of policies during student life, and 3) future: impact of policies for post-graduation. The final coding tree focusing on the nonimmigrant visa policy under U.S. immigration law that has affected international students is demonstrated in Fig. 2.

### Impact of July 6, 2020 Policy Directive

We investigated the impact of the July 6 Initiative on international students from various angles. From the enactment to rescindment (July 6 – July 14, 2020) of this policy, participants were asked how they first heard of it, what emotions they had, what actions they took, and what resources or services they needed.

#### Information source: how first heard

Participants recalled more than one information source that first informed them of July 6, 2020 Policy Directive. The majority of participants first heard of the July 6 Initiative online (e.g., Reddit) or on social media (e.g., Instagram, Twitter, WeChat, Facebook, WhatsApp). Then, some of them searched Google to get more information. They were in online groups (e.g., international student groups) to receive or share the latest information through those social media channels.

Ten participants stayed informed about politics through online newspapers or news podcasts and shared this information with others, even before the international student office at the university sent them a statement. One participant highlighted the importance of listening to the news:

As an international student, it’s definitely important if you care about your status to be informed about politics at all times. Throughout these four years, there’s not a single day where I don't listen to the NPR news podcast on Spotify for 10 minutes at least. (Participant #21, male, Iran, age 21)

Ten participants received a text message from an international student group chat. A few of them were in their home country when this policy was introduced. Those who do not read newspapers first heard this policy from their friends or family in their home country:

Do you know what’s funny? I did not find out about it, but my people, my friends and family in Vietnam saw that first and sen[t] [it] to me. I think, is kind of like a big thing because I don’t read newspaper[s]. . . . That is why people in Vietnam actually kn[e]w about it before me, and they [were] worrying for whoever they know that is currently [in] the US. (Participant #11, female, Vietnam, age 25)

#### Emotions

All participants had various negative feelings–stress, anger, frustration, confusion, anxiety, denial, betrayal, and unfairness–when they heard of the July 6 Initiative. Despite the fact that it was their right to stay in the United States, they were forced to register for at least one in-person class. However, almost all classes at the university were switched to an online delivery model due to COVID-19, and they did not have a choice but to register for the Physical Education (PE) in-person classes, such as swimming and soccer. They were uncertain whether they could go back to their home country to take time off from school or to continue their education by taking online classes remotely because they were afraid of the possible situation of not being allowed to return to the United States due to F1 visa cancellation. Besides all of those negative feelings, 2 participants worried about the situation of having no access to school materials due to restrictions on online connection or online learning management systems in their home country:

What if I go home and I cannot get back and the schools reopen? And I should get a visa again and everything will be like hell. It was really annoying. I was crying a lot. . . . There was something else that some countries that have problem[s] with I mean they are restricted to use some applications. . . . Being an international student during COVID was tough, really tough. (Participant #13, female, Iran, age 25)

When the policy was overturned a week later, all participants felt a huge relief and realized that there are people who care about them. Two participants were able to drop the unnecessary PE in-person class. The other participants were relieved but then had a complex feeling of bitterness because they could not avoid a stressful week due to this policy:

I found it funny and I was so stressed for a week. I mean honestly, I’m happy that it was overturned, but I was upset because I was really stressed for a week as a result of this policy. (Participant #6, female, Singapore, age 19)

#### Taking actions

During that one week of a chaotic, uncertain moment due to the July 6, 2020 Policy Directive, we wondered what actions participants took to solve the issue. One realistic solution was to follow the policy by registering for in-person classes and changing academic plans. Without knowing how many credits of one in-person class is at least needed, a few participants signed up for multiple in-person classes and waited for further announcements from the government, and 3 participants registered for one credit of PE class to maintain student status even though the PE requirement was already completed:

I didn’t want to do the [student visa] process all over again, because you never know if they [(administration)] won’t let you come back. So one of my plan[s] was to . . . try to get [in] an in-person class. So I was looking for any sports programs that will let me in, and I kn[e]w that there was a swimming class. (Participant #12, male, Cambodia, age 23)

Another solution that 6 participants tried was planning to go back to their home country to continue education remotely, but at least 2 of them had concerns about the cost of the flight ticket cost or returning to the United States:

I should just look for a ticket to go home, and I was really sad and angry, and I was crying and crying and said, ‘You know what, as far as I’ve been here, I have watched lots of news. I have seen lots of ups and downs of America, and I know they wouldn’t do this.’ (Participant #13, female, Iran, age 25)

They talked with their parents who financially supported their study and living expenses to notify what was happening and ask what action needed to be done. Some participants did not want to believe the policy; they contacted the International Student Office (hereafter referred to as “ISO” to indicate a university-wide office for supporting international students), friends, and read the news to check whether this policy is true. For example, Participant #9 tried to be more rational by reading diverse perspectives on this issue and was able to relax because of the following reason:

We didn’t want that to become a reality, so we didn’t even make it into a situation. What I did was just read about it more from different perspectives. Is it really worth it for the U.S. to get rid of international students when a lot of schools make a lot of money [from] international students? That was able to calm me down a little bit. (Participant #9, male, The Philippines, age 23)

Two participants considered transferring to universities in different states that provide the same program or more in-person classes, but it was unlikely to happen because they had to start over the program or had only one month to move to another university. Transferring to a university in their home country could be another option, but they were placed in a dilemma because studying abroad and earning a degree in the United States was their original goal:

I am in [an] athletic training program, so I was thinking of transfer[ing] to another state that has the same program but they told me, ‘You have to start from the beginning.’ I was like, ‘Okay, this is not the option.’ Then I thought about mov[ing] into Colorado or [a] near[by] state that had in person classes, but it was [already] July, and the Fall semester was starting in about a month. . . . I thought about going back to Japan to take a break, but I wasn’t sure if I can keep the F1 visa. I wasn’t sure even though I was trying to take some actions, there was too much uncertainty that I couldn’t do anything. (Participant #18, female, Japan, age 23)

The most proactive action for them to come to grips with this policy was to sign a petition from other universities. Five participants entered their names or posted the information on their social media to encourage others to participate in this action:

I started to sign a bunch of petitions. . . . To turn down the policy, and I was basically asking other friends, both in us in Japan to sign petitions and explain ‘Hey, this one is going to help me to keep continue studying [in the] U.S., so if you feel comfortable signing this petition, I will be greatly appreciate that’ and I was signing and just spreading these petition to all the friends. (Participant #10, female, Japan, age 22)

#### Needed resources or services

We wondered what kinds of resources or services should have been provided by the university to effectively overcome the July 6^th^ Initiative. Overall, participants did not receive satisfactory resources for support from their university. First of all, the majority of participants wanted more established mental health support and consulting service. For example, although the university provided a psychological consulting service to students, the number of appointments was limited to each student per year, and it did not provide a customized service that would help resolve unique situations that each student had. Because university counseling services have some limitations in understanding what situations international students have been through, participants and their friends ended up seeing a psychiatrist and receiving antidepressant medicine. One participant pointed out a lack of workforce for university counseling services:

I tried to reach out to one of the consult[ing] service[s] of our school once, but I think there are not enough human resources there to take care of it. Because now so many other services that our school provide[s] [are] closing down because of COVID, then it would be fair if they try to push more human resources into what they can still provide for us to make it fair. I think if they focus on student mental health and pushing more human resources into that service, it would be easier for international students. (Participant #11, female, Vietnam, age 25)

It was evident that international students could get lost in their studies due to this uncertain situation. Even an international student who had maintained top in classes lost the pace and desperately needed the mental and emotional support:

It became much harder to focus when eating, studying, researching, sleeping in the same single room. . . . I went from being in one of the top 3% of my class to probably feeling I’m barely understanding what I’m studying, which was very demoralizing. . . . As an international student, [I] just didn’t have the kind of support, that be the mental and emotional support like having a life here to kind of just help you pull yourself together. (Participant #1, male, Lebanon, age 25)

Second, some of them wanted to receive financial support for their classes, housing, and food from the university. Others did not see what alternative plans the university or ISO could offer. Participant #23 expected that ISO should have promptly provided correct information to international students, so they would be able to decide whether to stay in the United States or go back to their home countries. This participant at least wanted to hear from ISO was mental and emotional support messages even if they have no information to provide, such as ‘It is okay to stay positive and observe the situation at the moment’:

If we email International Student Office asking them all day, they will say, “Okay, we don’t know about that. We haven’t decided yet. We don’t have correct information for you.” . . . It just makes no sense to us because you’re International Student Office, right? So if you’re just saying “We don’t have correct information,” we can’t do anything right now. It will make us panic. Like we learned this from the news, it seems it will come true but you say “We don’t know anything,” but when you know what you can do, it’s too late. . . . Maybe they’ll say they can say things like “Just stay calm, even though it comes true, it will [take] a time period for you to decide whether you stay here or go back.” . . . We can learn lots of things online, [but] we’re not fully dependent on their information, but at least you [(ISO)] need to just know it’s okay. (Participant #23, male, China, age 25)

Third, some participants had a difficult time identifying and reaching out to a point person who can support them. For example, Participant #7 experienced a big communication gap with parents from his/her home country and did not know a person who can help with housing. This participant contacted the university police department to seek help but was not able to reach out to a point person even after continuous transferring calls. The information about the exact support channels for various situations was needed for international students:

I tell my parents that I am not comfortable [with] the place I am. . . . They basically assume it’s not true, or they assume that I’m exaggerating. I didn’t see much support and help from my parents. . . . Calling the cops was not a very successful experience because . . . they’re transferring me to someone else, someone else's transferring me to someone else. . . . I’m just leaving all that aside, knowing that no one’s here to help. (Participant #7, female, Iraq/UAE, age 18)

### Impact of policies during student life

The existing policies from student assistance programs at the university and the local levels did not support international students' adjustment in the United States during the COVID-19 pandemic. It was not clear for international students whether they could maintain an F-1 visa during unexpected political changes and the COVID-19 pandemic. Obtaining Curricular Practical Training (CPT) was challenging for them due to limited job opportunities.

#### Assistance programs

#### University level: ISO

The purpose of ISO provides advising services on immigration regulations to international students and assist them in their transition to life in the United States. However, participants had mixed feeling about the services provided by the university because during COVID-19 due to a lack of communication of the provided activities:

I feel our school lack of all their promotion and promotion of the resources. (Participant #2, female, Ukraine, age 20)

Many students heard information about policy changes through other outlets and wished to hear information specific to their visa status through the university directly:

I personally feel [ISO] could have done a lot more, holding a lot more Zoom sessions to make sure that the students are aware of what they [(Trump administration)] would be creating. . . . So hearing certain things that were directly from the university would give a lot more confidence to the students, rather than hearing from US agencies and other third parties. So the resource from the university was a bit lacking, I would say. It could have been a lot more better. (Participant #32, male, India, age 24)

Participants stated that they did not reach out for support to ISO because they had a negative experience previously and felt as if the counselors could not relate to their issues:

I don’t want to call [ISO] because they’re not going to help me. So they need to change that. And I think they need to also get some counselors who understand international studies specifically. (Participant #3, female, South Africa, age 25)

When asked about expected assistant programs or resources that have not been provided by the university, participants stated that they were aware of the existing programs but wanted to see more relevant programming helpful for their academic and professional development:

They [ISO] do host a lot of workshops or seminars to share with the students about the skills and help you success academically. They hosted a lot of activities where we can meet with native speakers here and do a lot of activities learn about other cultures [from] different countries. I think that’s really great in terms of helping us blending in and everything. The thing that I just want them to improve is actually the academic skill, greeting presenting and even small things [like] how to write an email to your professor or to your advisor. (Participant #11, female, Vietnam, age 25)

#### University level: wellness center

Although the university wellness center for students also provided remote counseling opportunities, participants felt that the service was lacking in regard to support for their mental health and access to the services. One of the students suggested that an online portal to aid with appointment scheduling would reduce the barrier to accessibility of their services:

I think [it would be great if I could] mak[e] an appointment with [counseling service name] [through] an appointment system where I can go on the web and book an appointment, as opposed to calling in. Calling in would just be more a lot more daunting, so I think it turns off a lot of people, so I really think the key is to accessibility. (Participant #26, male, India, age 26)

Another barrier mentioned by a different participant was the limited number of mental health support hours that international students could use per semester; thus, individuals’ underlying mental health conditions might not be fundamentally resolved:

They had already talked about the wellness center and how they provide mental health support. That being said, they do kind of limit the number of hours that you can access like therapy or mental health support. From the wellness center and I think, for me, that was the biggest deterrent from reaching out for help because I understood this was not a like something that could rely on 100%. It’s only a few hours per semester, and so it was probably like a bandage to something that I saw it, or I perceived as just bandage to something to a very big problem. Because of the hour limits. So that’s I think the hour limits on therapy or mental health available from the wellness center kind of help me perceiving and kind of in a less important. This is not as big of a help as it could be and I still have never reached out for therapy, the mental health Center from (University Name). (Participant #1, male, Lebanon, age 25)

#### University level: food pantry

In order to address food insecurity for students, the university provided a full-service food assistance program, supplying eligible students with fresh produce, non-perishable goods, and refrigerated items. Every calendar week, students were permitted to come to the pantry and receive food free of charge as long as they can provide their student identification cards and necessary forms. Three students stated that they were reliant on the university food support throughout the pandemic:

I’m really grateful for the food pantry and the fact that they’re open [during] the pandemic. Because I can’t work [due to F-1 visa status], I’ve been relying on the pantry a lot, so it’s been really helpful. When I go there once a week, relying on their stocks and stuff. (Participant #5, male, Myanmar, age 24)

I got a lot of notifications during the pandemic about the [university] pantry where you can get free food. (Participant #9, male, The Philippines, age 23)

#### External to university

International students faced the same adversity that domestic students faced, however, due to their visa status were ineligible for many federal and local assistance programs since it would question their ability to finance their education in the United States, which is a requirement to maintain their visa. When asked about the type of assistance that they wished to have received, the participants spoke of programs such as CalFresh and Financial Aid. Over 40% of the participants had a family income of less than $25,000 which would qualify them for many programs. International students are considered non-citizens that temporarily reside in the United States and that are not eligible for CalFresh benefits, while other non-citizen groups, such as those admitted for humanitarian reasons, and permanent residents qualify [46]. Even though participants felt they were contributing to society as much as their peers, they did not qualify for assistance:

There was one point how I feel unfair too, [considering the fact that] how much we pay [the university]. We’re not eligible for any grants [and] financial aid [when] all of my [American] friends were just leaving out of unemployment. I was just working 20 hours in a week and I still didn’t get any stimulus and then I’m not eligible for EBT [(Electronic Benefits Transfer)] food cards. So my parents [are] my only financial support. (Participant #2, female, Ukraine, age 20)

Just as Participant #2 mentioned, international students faced financial hardships due to the COVID-19 pandemic and were ineligible for financial support because they are not eligible for federal student aid due to their F-1 visa status [47]:

We didn’t get any help or any support from any financial help or support from state or federal resources at all. We just kind of left to just figure our lives on our own. (Participant #1, male, Lebanon, age 25)

#### F-1 visa

For students of foreign countries, a student visa, F-1 visa, is required to study in the United States. The student must be enrolled in a Student and Exchange Visitor Program (SEVP)-approved school, be enrolled full-time, and attend and pass all courses [48, 49]. If an international student decides to work in the United States on an F-1 visa, their designated school official (DSO) must authorize the employment or risk deportation, this includes CPT and OPT. Students also require DSO approval for changing majors, dropping courses, transferring schools, traveling outside the country, moving, extending graduation, or taking a break from school [48]. Participants spoke of the difficulties of acquiring their visa and entering the United States during COVID-19 and after the July 6, 2020 Policy Directive. A majority of the participants were first-generation college students who had no family in the United States; thus, figuring out how to maintain their visa status was left up to them to navigate through, without the help from their family. Those who had already acquired a visa and were studying in the United States prior to the COVID-19 pandemic expressed concerns over maintaining their visa status as an ongoing issue regardless of COVID-19:

I think it’s really stressful for international students because we always have to remember our visa. (Participant #3, female, South Africa, age 25)

One thing is definitely we need to maintain our full-time F1 status. (Participant #23, male, China, age 25)

Nine participants spoke of the stress that COVID-19 posed on ensuring that they were compliant with their visa. To maintain a[n] F-1 visa, students are only allowed to take one online class per semester, however with the transition to completely online learning, students did not have an option for in-person instruction:

It was a period of panic because the first tie the news comes out, it was like we’re moving online, and then it kind of hits us because you have to have 9 units in-person out of the 12 units [that] you’re required to take as a full-time student. (Participant #26, male, India, age 26)

Those students enrolled in a SEVP-approved school attempting to enter the country had difficulties acquiring an F-1 visa to study due to COVID-19 and the limited availability of appointments:

When we wanted to complete [approval of an F-1 visa], [the] visa embassy's [appointment] slots [were] not available for visa interviews. I had to wait for around three to four months and check every day whether there is an availability to book an appointment for my visa and there was not much availability in India, because these offices are basically operating not at a full scale, but at a partial scale so which made it very difficult for the students to basically book one single star, which [was] a lot more easier before [the] COVID-19 pandemic. (Participant #27, female, Taiwan, age 30)

Another participant struggled to enter the United States in September 2020 and had to prove that they were taking an in-person class at the airport of entry even though the July 6, 2020 Policy Directive had already been rescinded two months earlier:

Before I came to the States, I have to prove the custom [(U.S. Customs and Border Protection)] that I have one online course or the hybrid course but I almost failed to getting the stage, because when my flight was on September 6 right and then, when I come to the airport, it was at night and . . . then the custom asked me “do you have any proof that you have in-person, online course?” and [I] let him see my syllabus. But he still doesn”t believe in that, so he told me that “Can you just give me your professors” name or something so I can call her or just contact them?” (Participant #28, female, India, age 25)

#### CPT (Curricular Practical Training)

International students who have completed the first academic year in the United States can apply for CPT to fulfill an internship or practicum required by their academic program [50]. Even though the internship is part of the required curriculum for a particular major, there is additional long paperwork that students on an F1 visa need to fill out to procure an internship opportunity in the United States. Participants spoke of the stigma that international students face when applying for an internship or job; that stigma has worsened during the COVID-19 pandemic:

It’s harder for us to apply for jobs because we need our companies to sponsor our CPT. It’s a lot of paperwork. Even if we did qualify for the job, the HR [(Human Resource Department)] would sometimes just reject us because we’re international and didn’t want to go through the hassle of filing all the documents for us. . . . Since the pandemic happen[ed], internships ha[ve] been really hard to get. I mean even pre-COVID, internships for international students [are] already pretty hard cause you have to do the sponsorship and stuff. So right now, even with COVID happening, it’s almost impossible to get an actual internship. (Participant #14, male, Malaysia, age 22)

Participants wished for standardized rules to make it easier for them to know their eligibility to apply for CPT. One participant pointed out that every college has interpreted CPT requirements differently and felt difficult to understand which rule is correct:

Some colleges have one day CPT [training] [that] you can start doing the part time or full time job when you are first joining the college. Some colleges have [a] one year rule, or some students can do only one internship. I don’t understand why there [are] different requirements to CPT. If there is like one rule for the CPT for all the colleges and all the courses that would make student life easier. (Participant #19, male, India, age 26)

### Impact of policies for post-graduation

As a short-term goal, 18 participants wanted to successfully graduate and stay in the United States. As a long-term goal, two trajectories were mainly mentioned. One group wanted to study further for an advanced degree (master's, doctorate, or medical degree). The other group wanted to find a job in the United States. They mentioned the geographic advantage of being in the San Francisco Bay Area to work at their dream company and achieve their clear goals in the future:

I’m a Computer Engineering major and I chose that major because I want to be involved in designing CPUs and GPUs, so my dream would be working at either (big company name 1) or (big company name 2). . . . If quantum computers were to become mainstream in my lifetime, I would like to be able to work on them, because Silicon [Valley] is reaching its absolute limit right now in being the size of an atom and we cannot go further than that, then we’re going to have to make quantum computers mainstream and so that’s going to be equivalent to when we first had Silicon-based computer. It’s going to be the next big thing, and I want to be in on that. (Participant #20, male, Malaysia, age 24)

International students receive the duration of status (D/S) on the admission stamp in their passport, allowing them to remain in the United State as long as maintain their nonimmigrant student status, and it can be extended by the end of their OPT [51]. Unless they change their status with a new program of study or with a job offer, they have 60 days to depart the United States [51]. For those who pursue an advanced degree, a new F1 visa would be required. For those who pursue a job, there are various temporary (nonimmigrant) worker visa classifications available to lawfully work in the United States [52]. Among those nonimmigrant classifications, H-1B applies to international students who would graduate and have a job in the United States. After 12 months of post-completion OPT (for all international students) and an additional 24-month STEM OPT extension (for eligible international students), an H-1B visa and then permanent residency is needed for those who would want to stay in the United States. However, several challenges remain in obtaining an H-1B visa and permanent residency.

#### OPT (Optional Practical Training)

Students who are studying in the United States can participate in Optional Practical Training (OPT) before completion (pre-completion) and/or after completion of their academic studies (post-completion) [53]. Those who have completed their degree are given up to 12 months of OPT related to their study at each educational level [53]. Participants expressed concerns regarding OPT policy during their enrollment in college before and due to COVID-19. The students spoke of barriers to qualifying for OPT and securing a job after graduating due to the timeline, restrictions, and stigma behind hiring an international student. Participants were worried about the difficulty of getting approved for OPT due to COVID-19 because of the increased processing time, the reduced job market, and their country's status. During that time, some OPT applications took up to 5 months to get approved, which means international students would lose a maximum of 5 months out of 12 months of the post-completion OPT employment authorization if they do not apply for OPT in advance:

For OPT right now, because of COVID, I’ve heard that some are even taking five months to get approved. A lot of countries right now are still on lockdown, like myself, we cannot go back [to my home country] even if we want to. . . . If we don’t get approved for OPT, we’re basically out of status [in the U.S.]. And even if we get approval [of] OPT, because of the pandemic and the current job market, . . . we’re screwed. We’re supposed to go back [due to July 6 Initiative], but at the same time, because of COVID and the lockdowns we cannot go back, so what are we supposed to do? (Participant #5, male, Myanmar, age 24)

It was a difficult time-consuming process for international students to prepare all application materials and wait for OPT authorization. In addition, they felt that they are not welcomed in the U.S. society and had limited work opportunities for international students:

I never feel really welcome to be [an] international student [because] there’re limited job opportunities. To get [an] internship you have to do OPT and 10,000 steps before you even get the position. (Participant #2, female, Ukraine, age 20)

As an international, if you want to go through the OPT process, . . . it might as well be your own lawyer. It’s a whole crazy process to go through, if you really want to be successful today in the U.S. (Participant #21, male, Iran, age 21)

There was another hurdle to overcome for those who are authorized for post-completion OPT; if they are unemployed for 90 days from the OPT start date, they have to leave the United States. Participants expressed difficulties in finding job opportunities within those short period, especially during COVID-19:

Only internship[s] [are available], not the full-time position. . . . OPT is only one thing that guarantee[s] my current stat[us] in the U.S. I want to stay here, but how [do I] get a job in three months? It’s quite [a] tough time. [It’s] quite challenging for me [to] graduate this semester. (Participant #4, male, Thailand, age 32)

#### STEM (Science, Technology, Engineering, and Mathematics)

Graduates who have completed a degree in STEM fields are eligible for an extension of 24 months of their post-completion OPT [54, 55]. For those who are in the STEM program, they expected that the result of the 2020 United States presidential election positively impacted their plan to stay in the United States. Participant #23 shared expectations of maintaining the policy of a maximum three-year of OPT for STEM fields:

I’ll say just keep it [(STEM)] as what it [is]. I’m in the STEM program. If you [(Biden administration)] don’t change our three-year for OPT, that [means] a lot [to] me. I changed my major to data science [and] will have three-year OPT. . . . You should get prepared, you should get everything done [during] this time, so actually for me, [it is] the greatest news. (Participant #23, male, China, age 25)

#### H-1B visa

The first challenge that international students faced was the uncertainty of receiving a job offer in the United States during the COVID-19 pandemic. Second, even if they accept a job offer, there is no guarantee that they would receive an H-1B visa because USCIS conducts random selection from the entire H-1B registrations each year, called the H-1B visa lottery. Most recently, for fiscal year 2022, USCIS selected about 43% (a total of 131,970 H-1B registrations) of 308,613 H-1B registrations through three random selection processes [56]. Only those who are selected during one of those processes are eligible to file H-1B cap-subject petitions to receive an H-1B visa [56]. Although the H-1B classification has a numerical limit (cap) of 65,000 new visas or statuses each fiscal year, there are additional 20,000 petitions as an advanced degree exemption for those who earned a master’s degree or higher at a U.S. institution of higher education [57]. In addition, H-1B petitions for those who are employed at “an institution of higher education or its affiliated or related nonprofit entities, a nonprofit research organization, or a government research organization” are exempted from the cap [57–59]. One participant did not understand why the university does not provide detailed information about an H-1B visa:

If you work for a nonprofit, you don't have to enroll in the H-1B lottery. I don't know why our school or nobody mentioned this. They just say that you're going to have one year OPT and after that, you're going to attend this H-1B lottery when you have 30% of being able to stay in [the] U.S. and get a job. (Participant #11, female, Vietnam, age 25)

Ten participants had concerns about changes in visa requirements whenever an administration is inaugurated and could not set a clear future goal in the United States due to the uncertainty of whether they can stay or not. Their plans to continue living in the United States heavily rely on the administration's immigration policy:

I don’t know if this current administration will change the H1 visa or the work visa for me, because if they change to make it harder to [get a job], then I will go back to my country. But other than that, I want to have a job here, but . . . I don’t feel certain to work [in the] U.S. because of changing visa requirements. . . . I don’t have much career goal[s] [off] the top of my head now. (Participant #17, male, Vietnam, age 20)

A few participants who were keen on U.S. immigration reform during the Biden Administration were aware of Biden’s proposed changes of reversing Trump-era restrictions on immigration to the United States [60–62] and saw the positive opportunities of pursuing their dreams in the United States. Since President Biden took office, “a fair, orderly, and humane immigration system” has been built [63, 64]. For H-1B visas, the Biden administration withdrew the "Strengthening of the H-1B Nonimmigrant Visa Classification" rule that increased the denial of petitions [65] and delayed the implementation of “Strengthening Wage Protections for the Temporary and Permanent Employment of Certain Aliens in the United States” rule that would have raised prevailing wages of H-1B recipients [66–69]. In addition, the administration rescinded the proposal for the elimination of the H-4 Employment Authorization Document (EAD) program [70, 71]; Vice President Harris advocated for spouses of H-1B workers holding H-4 EADs [72] and was inspirational to some participants in regards to promoting women empowerment:

The one thing which I got to know from the new administration is about something related to H-1B visa. . . . I think the percentage of H-1B given to international people has increased in Joe Biden’s administration. . . . Kamala Harris, [was] born here and she’s the first female Vice President [of] the US. I think that has a lot to do with the wom[a]n’s figure. (Participant #31, male, India, age 23)

#### Permanent residency

In the long run, participants can pursue applying for lawful permanent residency (a Green Card) as an immigrant worker [73, 74]. The Biden administration proposed increasing the number of employment-based green cards, using unused visa slots from previous years, allowing spouses and children of H-1B holders to receive green cards, and removing the 7% per-country ceiling [61, 75, 76]. Some participants see their positive future in the United States with the hope of having more confidence and passion:

So, passion, that thing was definitely I should have just kept it to myself, but the more dominating motivation would be trying to get the capital first. Trying to establish a green card or an H1 visa here and from there when I have capital, I can do much more things, whether it's to pursue my passion, help my family out. (Participant #14, male, Malaysia, age 22)

## Discussion

The results show that the nonimmigrant visa policy has directly impacted international students’ psychological adjustment and well-being in the United States. In addition, many participants expressed financial challenges, especially during the COVID-19 pandemic. They were not allowed to get paid for non-school related work due to the F-1 visa’s employment policy [77]; since federal financial aid or student loan is not available for international students, parents' financial support might be the only way for them to pay the tuition and living expense unless they have a good external scholarship. Those external scholarship opportunities are only given to competitive students. The COVID-19 pandemic and the July 6, 2020 Policy Directive increased stress for international students on acquiring and maintaining their visa with the changes in instruction, lack of communication, and reduced office hours. Many participants had mixed feeling about the support by ISO during the policy changes and were unable to fully utilize what was offered since they felt that communication was lacking. International students were able to take advantage of some university programs, like the food pantry and the wellness programs, but stated barriers due to availability and limitations. Some participants wanted to receive continued support beyond graduation from the university. Regarding visas, for instance, ISO staff are responsible for an F-1 visa, CPT, and OPT, but may have no obligations to provide every detailed information about H-1B information. We observed the different scope of expectations between students and universities; while the role of a university could be typically explained as providing education and offering diplomas and certificates, some participants wanted the university’s continuous advice beyond OPT, until they successfully get a job in the United States. Participants expected that the university should provide strategies for career advancement and visa options after OPT, such as H-1B and any other possible longer-term visa. While almost all of the participants were keen on politics, one participant was indifferent to the result of the 2020 presidential election thinking his/her plan for STEM would not be affected. However, it is apparent that depending on the direction of the administration, more STEM opportunities can come. Recently, the Biden administration has expanded the number of majors in science, technology, engineering, or mathematics (STEM) fields to retain and attract STEM students [78, 79].

COVID-19 and nonimmigrant visa policy have become closely intertwined, causing a growing uncertainty in the academic performance and legal status of international students. This study contributed to addressing gaps in the current literature about the specific impacts on, and needs of an international student group in a public health crisis as follows. First, it provided significant insights into international students' right to study in a safe learning environment. Second, it highlighted the urgent need of scaling up evidence-based mental health interventions and preventive strategies to address the mental health outcomes of an international student group during unexpected future long-lasting disruptions. Lastly, it serves as a base for future studies that recommend desirable immigration policy and practice for strengthening international education leadership in the United States. Future studies should concentrate on investigating the importance of the positive association between international education and the United States' global leadership and economic strength.

### Limitations

The findings in this study are subject to at least two limitations. First, this study was conducted through convenience and purposive sampling methods, which would have limited external validity [80]. All participants were from one university in the San Francisco Bay Area, California, which was conveniently accessible to the authors, may not be representative of the international student populations at large in the United States. Although this study was not able to recruit participants exactly proportional to the leading places of origin of international students [81], at least it reflected the pattern that the majority of international students are from continent Asia [82] by having a great number of Asian participants in the in-depth interviews. Second, due to the absence of in-person one-to-one interaction for an interview, it could be possible that trust-building between an interviewee and an interviewer might not be fully established. Although we asked an ice-breaking question, “Do you have any good plans for this summer?” to a participant before officially beginning an interview, conducting an online in-depth interview might not be a comfortable environment for some participants to share their experiences and thoughts with an open mind. Lastly,

## Conclusions

The international student group in the United States has been largely affected by the policy changes due to the COVID-19 pandemic. Uncertainty of policy change due to COVID-19 and presidential transitions–the July 6, 2020 Policy Directive, policies during the study in college (student assistant programs, F-1 visa), and policies for post-graduation (OPT, H-1B, and permanent residency)– negatively impacted international students’ psychological well-being and adjustment because they experienced the difficulty of making a plan to stay in the United States. Historically, international students have shown economic contributions to the United States, become an essential workforce where specialties are in high demand, and added unique academic and cultural values to the school campus and local communities. Future policies at the school, local, state, and federal levels can offer essential insights into supporting international students' life and improving their health equity and mental health to assist them to contribute to the economy, culture, and society of the United States.

## Data Availability

The datasets generated and/or analyzed during the current study are not publicly available due to the confidentiality of participants’ information but are available from the corresponding author on reasonable request.

## Abbreviations

CPT: Curricular Practical Training
OPT: Optional Practical Training
ISO: International Student Office
STEM: Science, Technology, Engineering, and Mathematics
